# A Methodologic Approach for the Selection of Bio-Resorbable Polymers in the Development of Medical Devices: The Case of Poly(l-lactide-*co*-ε-caprolactone)

**DOI:** 10.3390/polym10080851

**Published:** 2018-08-01

**Authors:** Alberto Cingolani, Tommaso Casalini, Stefano Caimi, Antoine Klaue, Mattia Sponchioni, Filippo Rossi, Giuseppe Perale

**Affiliations:** 1 Institute for Chemical and Bioengineering, Department of Chemistry and Applied Bioscience ETH Zurich, Vladimir-Prelog-Weg 1-5/10, 8093 Zürich, Switzerland; alberto.cingolani@chem.ethz.ch (A.C.); tommaso.casalini@chem.ethz.ch (T.C.); stefano.caimi@chem.ethz.ch (S.C.); antoine.klaue@chem.ethz.ch (A.K.); 2 Industrie Biomediche Insubri SA (IBI), Via Cantonale 67, 6805 Mezzovico-Vira, Switzerland; 3 Biomaterials Laboratory, Institute for Mechanical Engineering and Materials Technology, SUPSI—University of Applied Sciences and Arts of Southern Switzerland, Via Cantonale 2C, Galleria 2, 6928 Manno, Switzerland; 4 Department of Chemistry, Materials and Chemical Engineering “G. Natta”, Politecnico di Milano, 20100 Milan, Italy; mattia.sponchioni@polimi.it

**Keywords:** electron beam, ethylene oxide, medical devices, polymers, sterilization

## Abstract

In the last decades bioresorbable and biodegradable polymers have gained a very good reputation both in research and in industry thanks to their unique characteristics. They are able to ensure high performance and biocompatibility, at the same time avoiding post-healing surgical interventions for device removal. In the medical device industry, it is widely known that product formulation and manufacturing need to follow specific procedures in order to ensure both the proper mechanical properties and desired degradation profile. Moreover, the sterilization method is crucial and its impact on physical properties is generally underestimated. In this work we focused our attention on the effect of different terminal sterilization methods on two commercially available poly(l-lactide-*co*-ε-caprolactone) with equivalent chemical composition (70% PLA and 30% PCL) and relatively similar initial molecular weights, but different chain arrangements and crystallinity. Results obtained show that crystallinity plays a key role in helping preserve the narrow distribution of chains and, as a consequence, defined physical properties. These statements can be used as guidelines for a better choice of the most adequate biodegradable polymers in the production of resorbable medical devices.

## 1. Introduction

Biodegradable polymers have become, in the last decade, a major base material for the development of many different bioresorbable medical devices [1,2,3]. Thanks to their intrinsic characteristics and chemical and physical nature, they perfectly match with this kind of specific application, as they ensure high performance, complete biocompatibility, and tunable resorbability at the same time [4,5,6]. 

Indeed, playing with the initial composition of polymers and their molecular weights, mostly derivatives of polylactic acid (PLA), of polyglycolic acid (PGA) and of polycaprolactone (PCL), and/or their copolymers and blends, enables them to perfectly combine desired mechanical characteristics with a fully bioresorbable product [7,8]. Specifically, they properly address the big disadvantage of the post-healing surgical intervention for devices that need to be implanted into the patient’s body [9], because, upon degradation, they will be naturally eliminated by the organism without necessity of direct removal [10,11,12].

These considerations, coupled with the intrinsic ease of processing and the production of polymer-based building blocks, both in the form of individual polymer chains [13] or in the micro and nanoparticles fashion [14,15,16], make them a very robust path towards the development of advanced medical devices and controlled drug delivery systems. Indeed, many different applications have been properly exploited and developed, from biodegradable suture systems [17], to polymer capsules for drug delivery application [14], hydrogels [18] for controlled drug release, and scaffolds for cells in tissue engineering [19]. As mentioned before, the great advantage that all of the aforementioned products have in common is that they will naturally disappear from the patient’s body in reasonable and controllable time after the implantation, leaving minimal traces and small impact [1]. 

When dealing with implantable medical devices, it is very important to point out that, in industrial practice, product formulation and manufacturing need to follow specific procedures. Indeed, once the desired inputs are defined (i.e., mechanical properties and degradation profile) accurate selection of the base material needs to be done. This has to take into consideration not only the characteristics of the pristine base polymer but also the way they will be affected by all the manufacturing and post-processing steps. 

As a matter of fact, processes such as thermoforming, injection molding, extrusion and in general all of those that are performed at medium to high temperature and/or applying mechanical stress can significantly alter the polymers features [20]. Moreover, though some steps might be product dependent, certainly terminal sterilization represents in this sense not only a major point, but also a crucial, necessary, and compulsory passage in the production of every implantable medical device [21,22]. Specifically, it aims at the inactivation of any microbiological contaminants that might be present on the final products. Moreover, although preparation conditions might be perfectly in accordance with quality management system guidelines (i.e., ISO 13485-2016), the finite outputs can be considered sterile only if they are free from any viable microorganisms [23,24]. The necessity on relying on a fully validated and fixed protocol, ensuring reliable and reproducible performances, therefrom derives. 

The most widely used industrial terminal sterilization techniques imply either steam, ethylene oxide (EtO), γ or electron beam irradiation [25]. Regarding polymer-based devices, not all of the aforementioned possibilities are available as, for example, steam and γ irradiation frequently cause excessive degradation and changes in physical or mechanical properties, which can be detrimental for intended performance, especially in terms of degradation rates and times and also device shapes and dimensions [24,25]. Thus, the most conventional solutions in this sense involve electron beam radiations and exposition to alkylation agents (e.g., ethylene oxide) [23].

Apart from the composition and the molecular weights (*M*_w_, *M*_n_, *PD*), that clearly have a role in polymer response to all manufacturing and post-processing operations [20,26], and are generally taken into consideration in product formulation, often also other crucial physical parameters (*T*_g_, *T*_m_, crystallinity) [27,28], might be of great interest, though many times neglected. A superficial characterization generally leads to major issues when different raw material suppliers or formulations are required, especially because the final response to the whole production processing, including sterilization, might not be identical [29]. 

In this work we decided to study the effect of the two most common different terminal sterilization methods for resorbable biopolymers (EtO and electron beam) on two commercially available poly(l-lactide-*co*-ε-caprolactone) with equivalent chemical composition (70% PLA and 30% PCL) and relatively similar initial molecular weight, but with different chain arrangements and crystallinity. 

These polymers find application in the production of devices for hard tissues fixation and regeneration [30,31,32]. As a matter of fact, it resulted that crystallinity, indeed, plays a role in the response of these apparently equivalent polymers to different sterilization techniques, helping in preserving the narrow distribution of chains and, as a consequence, defined physical properties. Furthermore, we employed independent experimental data from the literature in order to quantify the impact of the sterilization on the actual degradation rate through mathematical modeling. 

As an outcome, we identified two major points to be taken into consideration in the formulation of polymer-based medical devices: first, the importance of establishing the impact of the selected terminal sterilization methodology on the polymeric material itself; second, the characterization of the base polymer cannot simply focus on the molecular weight and chemical composition but needs to be extended to other physical parameters. These statements can be used as guidelines for the usage of biodegradable polymers in the production of bioresorbable medical devices.

## 2. Materials and Methods 

### 2.1. Materials

The following chemicals have been used as supplied, without further treatment: poly(l-lactide-*co*-ε-caprolactone) purchased as PLC 70 from Purac-Corbion (Amsterdam, The Netherlands), named from now on polymer P; poly(l-lactide-*co*-ε-caprolactone) purchased as RESOMER^®^ LC 703 S (Evonik, Essen, Germany), named from now on polymer E; tetrahydrofuran (THF) and deuterochloroform (CDCl_3_) (Sigma Aldrich, Steinheim, Germany). 

### 2.2. Terminal Sterilization Procedures

Sterilization of a product is properly intended to remove all the living microorganisms contained in or adhering to it, including their resistant dormant bodies, such as spores [23]. When dealing with medical devices this passage is often referred to as terminal, because it occurs on the fully finished product, already packed and ready to be sold. A major consequence of this is the impossibility of analytically testing again each individual device as it would mean removing the packaging and indirectly invalidating the previous sterilization, making all the previous operations useless. Therefore, it is necessary to imply a consolidated, reliable, and validated protocol, based on routinely monitored procedures and equipment, which must ensure full sterilization and limited damage on the product. Thus, the selected sterilization conditions must take into consideration, on one hand the number and resistance of microorganisms in the environment in which the treatment is performed and on the other hand the necessity of limited interference with the initial desired characteristics of the final product. All sterilization protocols involved in this work have been conducted in accordance to the aforementioned requirements and are the actual ones currently in use for conventional protocols in the production of biodegradable polymer-based medical devices. 

#### 2.2.1. Ethylene Oxide Processing

Sterilization via EtO has been performed on both P and E polymers, following a validated protocol, in accordance with the guidelines described in detail in ISO 11135. These procedures take into consideration manufacturing conditions, construction materials and product design, including geometric variability and packaging characteristics (i.e., the container must have good EtO permeability). 

The physical performance qualification allows the verification of the cycle reproducibility as well as the evaluation of the cycle impact on the product, packaging functionality and safety. Specifically, the samples, packed into a sealed, sterile plastic bag and aligned in a rack within a carton box are firstly pre-conditioned for a time range of 105–170 min at a temperature range of 48–52 °C. After the cycle starts, it first reaches vacuum within a time range of 60–120 min at a temperature in the range of 48–52 °C, then the product is exposed to EtO in gas form for a time of 345–375 min, consistently at the previous temperature and a humidity higher than 60%. The concentration of the gas in this phase is in the range of 320–322 g/mc. This last passage is performed at least three times. Finally, a degassing step of 1425–1455 min at temperature of 41–51 °C is performed.

#### 2.2.2. Electron Beam Processing

Electron beam sterilization has been applied following a procedure approved and described in the standard ISO 11137. Samples, sealed in glass vials, closed with a plastic stopper and an aluminum cap and housed in a carton box, were passed through the chamber for a few minutes at 47.4 °C and invested by a radiation dose in the range of 25–30 kGy. As the beam hits the samples, electrons penetrate the cardboard box and all the samples in their individual packages inside the carton [23]. This ensures that harmful microorganisms are completely inactivated. More specifically, as the electrons penetrate the products, the radiation dose diminishes, therefore, in reality, less radiation leaves the box then entered. Thus, the boxes are usually turned over and irradiated again from the opposite side in order to get a relatively uniform dose. 

### 2.3. Analytical Methods

Polymer samples have been characterized both before and after each sterilization procedure using the analytical techniques presented as follows.

#### 2.3.1. Gel Permeation Chromatography

Weight-average (*M*_w_) and number-average molecular weight (*M*_n_) values and molecular weight distributions (*M*_w_/*M*_n_) values of the polymers were evaluated using a Jasco LC-2000Plus gel permeation chromatograph (GPC) equipped with a refractive index detector RI-2031Plus (Jasco, Oklahoma City, OK, USA) using 3 Agilent (Santa Clara, CA, USA) PLgel columns, 5 × 10^−6^ m particle size, 300 × 7.5 mm (*M*_w_ range: 5 × 10^2^ to 17 × 10^5^ g·mol^−1^). THF was chosen as eluent at a flow rate of 0.5 mL·min^−1^ at 35 °C. The GPC samples were injected using a Jasco AS-2055Plus autosampler. The instrument was calibrated using polystyrene standards from 580 to 3,250,000 Da (Polymer Laboratories, Church Stretton, UK). Probes have been run in duplicates, showing full consistency and overlapping of the data. 

#### 2.3.2. Differential Scanning Calorimetry

Differential scanning calorimetry (DSC) measurements were conducted using Q1000 Differential Scanning Calorimeter (TA Instruments, New Castle, DE, USA) using 40 μL crucibles in aluminum and a heating and cooling rate of 5 °C·min^−1^ in a nitrogen atmosphere (*T* ranges: 0–200 and 0–300 °C). Probes have been run in triplicates, showing full consistency and overlapping of the data. 

#### 2.3.3. X-ray Diffraction

The crystal structure was investigated by the grazing incident X-ray diffr PLgel columns, 5 × 10^−6^ m particle size, 300 × 7.5 mm action (XRD) (Malvern Panalytical, Malvern, UK) technique with Cu *K*α radiation in Bragg-Brentano configuration with the scanning angle of 3°. Probes have been run in duplicates, showing full consistency and overlapping of the data. 

#### 2.3.4. Nuclear Magnetic Resonance (^1^H-NMR)

Nuclear Magnetic Resonance (^1^H-NMR) was run on samples prepared by dissolving 50 mg of species of interest in 3 mL in CDCl_3_ and analyzed with H-NMR 300 MHz from Bruker (Billerica, MA, USA). Probes have been run in duplicates, showing full consistency and overlapping of the data. 

## 3. Results and Discussion

### 3.1. Prystine Polymer Characterization

**Figure 1:**
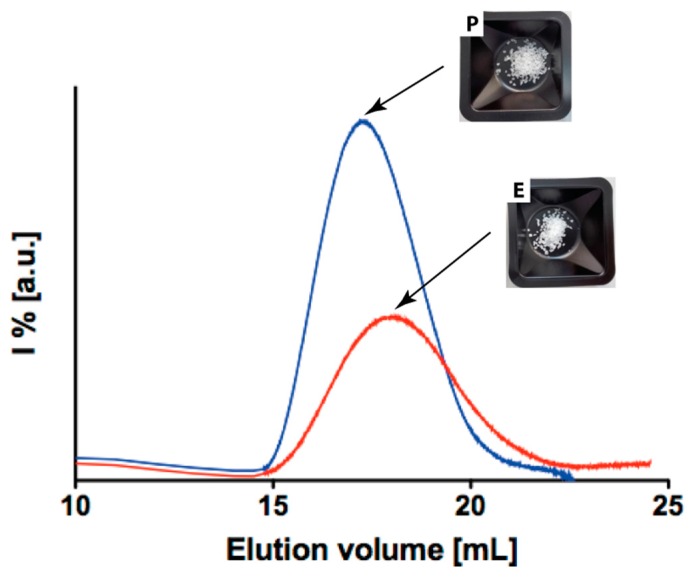
Gel permeation chromatograph (GPC) chromatograms of P (blue line) and E (red line). Picture of the two pristine polymers as removed from the supplier’s packaging.

In this section, the characterization of the two pristine polymers is reported. In Figure 1 the GPC chromatograms for both polymer P and E are visible and the characterization results, including also the other analytical techniques, are presented in Table 1. As is evident, their initial molecular weight is relatively high in both cases, and the NMR spectra (Figure S1 in S.I.) shows full consistency in their composition, which is confirmed equal to the one declared by the suppliers of 70% PLA and 30% PCL (full data in Table 1). 

**Figure 2:**
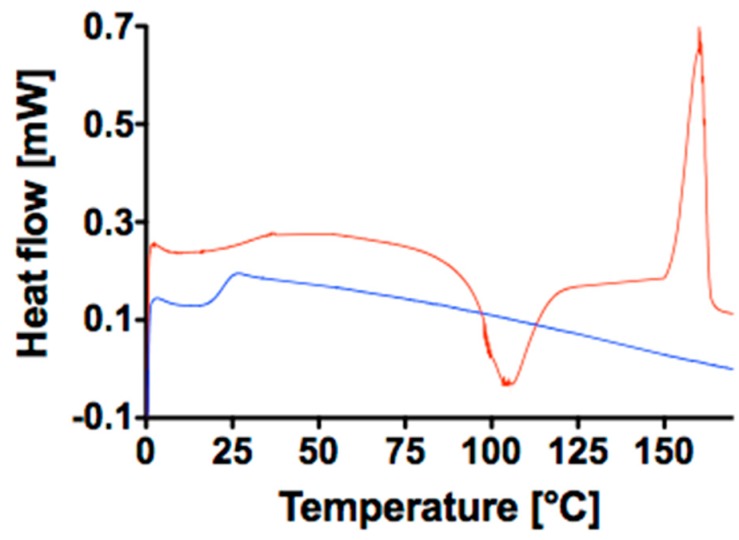
DSC curve of P (blue line) and E (red line).

On the other hand, still NMR, DSC (Figure 2) data, and XRD enable us to identify considerable differences between the two polymers (data in Table 1). As a matter of fact, E polymer has a crystallinity degree of 65% whereas P is around 35%. This statement is well supported by the NMR and DSC curve of Figure 2. Indeed, polymer E presents a well for the crystallization temperature, at 106 °C, and a defined melting point at 160 °C, whereas polymer P does not show any of the aforementioned detectable points and the shape of the curve clearly resemble one of the amorphous polymers. A similar conclusion can be drawn when looking at the XRD measurement (Figure S2 in S.I.) that also confirms the partial crystalline nature of E and the amorphous one of P. Indeed, from the Scherrer equation we calculated full width at half maximum (FWHM) and average grain size (*L*_hkl_) [33] and the more crystalline nature of E sample is evident. Therefore, though the two polymers might look similar from the composition and up to certain extent also from the molecular weight perspective, it is nonetheless reasonable to expect different physical behavior and response to the sterilization protocols in dependence of their crystallinity.

### 3.2. Ethylene Oxide Processing

**Figure 3:**
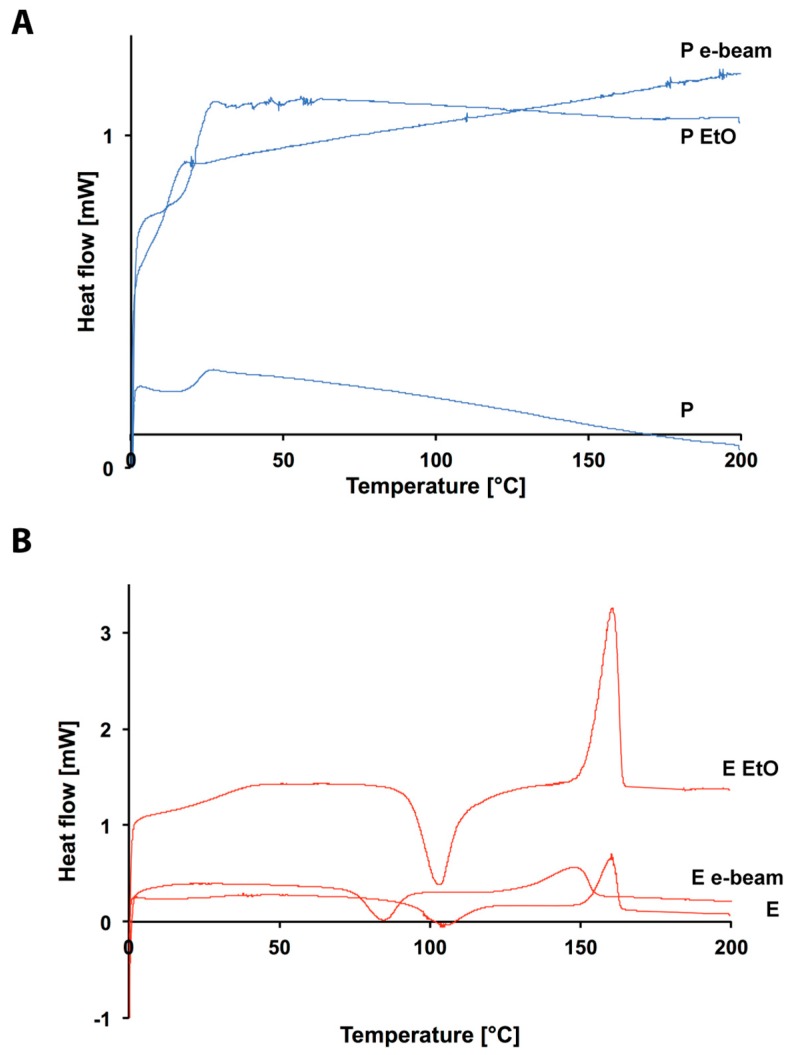
DSC curves of: (**A**) P, P EtO and P e-beam (blue lines); (**B**) E, E EtO and E e-beam (red lines).

**Figure 4:**
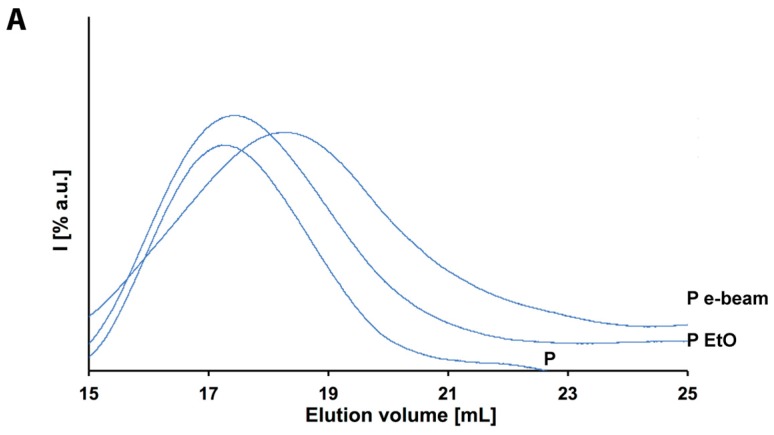

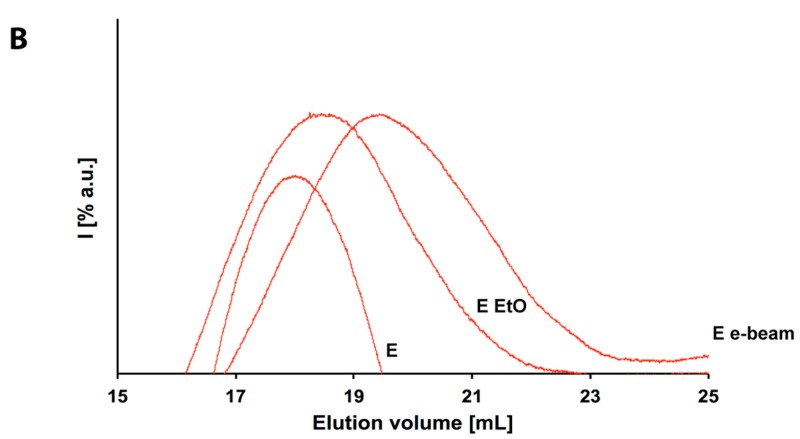
GPC chromatograms of: (**A**) P, P EtO and P e-beam (blue lines); (**B**) E, E EtO and E e-beam (red lines).

Though relatively expensive, EtO processing is widely used for the sterilization of medical devices and surgical instruments and it basically consists of a controlled exposure of the products to EtO in gaseous form, in a sealed chamber. The high diffusivity of EtO, coupled with its high reactivity, is of major importance for the inactivation of microorganisms. In fact, EtO can penetrate selected packaging and access all the exposed surfaces of the product. Moreover, it works as an alkylating agent for protein essential for cell reproduction, DNA, and RNA. This way, it prevents normal cellular metabolism and the ability to reproduce of the affected microbes, which becomes nonviable [24]. In general, the chemical species targeted by EtO are not included in most of the medical devices’ composition, therefore, their exposure to EtO should have very little or no impact on them, independently of their physical characteristics. On the other hand, the whole process is performed at mid-high temperature and relatively high humidity, both parameters that might affect the polymer initial fashions. Indeed, though not great changes are recorded in the physical characteristics of the polymers (DSC in Figure 3, NMR in Figure S3 and XRD in Figure S4 in S.I.), still a statistically relevant albeit small reduction of the molecular weights is recorded for both of them (Table 1). As a matter of fact, the *M*_w_ of polymer E moves from 119,609 to 96,000 Da, with a change in *PD* of 0.013, whereas the one of polymer P shifts from 172,746 to 159,413 Da with a *PD* variation of 0.04 (Figure 4). Though these changes might not be an effect of the EtO directly, it is anyhow rather important to record the inevitable degradation effect given by the whole sterilization protocol.

### 3.3. Electron Beam Processing

Electron beam irradiation processing (e-beam) is commonly used in the sterilization of medical devices and in general represents a much faster and cheaper solution in respect to EtO processing. The procedure involves irradiation of the products with a high-energy electron beam, which ionizes in a controlled way the hit samples. The bombardment results in a cascade of free electrons through the material domain, which, when interacting with surrounding molecules, generates free radicals. These last species induce breaks in the DNA double helix, preventing replication and expression, therefore enabling sterilization effects [23]. Due to its mechanism of action, it is important to limit the duration of the whole irradiation to the minimum (generally just few minutes), otherwise great damage on the final products, such as polymers embrittlement, oxidative damage, and color change might occur [34,35]. Indeed, especially for polymer based devices, ionizing radiations exhibit an important side effect that affects their performance, such as a decrease in both number and weight, average molecular weight, and modification of the chains distribution and conformation. 

Such an effect is evident already at the minimal radiation dose (the “overkill dose”) that ensures sterilization, equal to 25 kGy [36]. Indeed, the free radicals and ions can also lead to recombination reactions, hydrogen abstraction or cross-linking reactions [37]. Moreover, if radiation energy is higher than intramolecular forces, unzipping reactions (i.e., depolymerization reactions) can occur. Generally speaking, the molecular weight decrease is proportional to the radiation dose [37,38,39]. Moreover, the specific trend depends on material composition (which determines the reactions pathways), degree of crystallinity, and sterilization environment (i.e., temperature and the presence of air, since oxygen molecules enhance the molecular weight decrease). This phenomenon has an important impact on the final behavior of devices made of aliphatic polyesters, which in general reflects on the mechanical properties of the finite device. Indeed, though the Young modulus decreases very slightly, the elongation at break diminishes dramatically, following a dose-dependent trend. In addition, radiations also influence the glass transition temperature, melting temperature, and the degree of crystallinity [38,40,41,42]. 

In general, the two main reactions that take place during irradiation are chain scission and cross-linking [26,28,38,43]. The ratio between methylene and ester groups CH_2_/COO is a very important parameter, because it discriminates the structure of the material after irradiation dose [38]. In particular, for high values of CH_2_/COO ratio cross-linking is the dominant kinetic mechanism, while at low values polymer degradation mainly occurs. 

In particular, polyglycolic acid exhibits the lowest CH_2_/COO ratio and experiences degradation while irradiated; cross-linking reactions appear only after very high radiation doses. Regarding the considered example of poly(l-lactide-*co*-ε-caprolactone), as one can see from Table 1, a clear decrease after irradiation in molecular weight *M*_w_ for both polymers E and P is observable. Indeed, the final values are 51,814 Da for E and 133,265 Da for P. In this sense, this is a huge difference in respect to the previously discussed EtO sterilization treatment. Moreover, though the variation of polymer P might appear to be smaller than the case of E, this result has to be taken into consideration in light also of the *PD* values of both distributions. As evident from Table 1 it varies considerably between the two polymers: E shows a variation of 0.113 whereas P one of 0.48. This means, that in the case of P much more oligomers are produced upon irradiation so that the distribution considerably enlarges. Therefore, clearly it would be more difficult, from a process perspective, to rely on the selected initial properties of the pristine material. On the other hand, polymer E almost preserves its intrinsic polydispersity, making the effect of the treatment on the final properties of the finished device more easily predictable. The difference that is observed within the two ionized polymers can be explained by the mechanism of action of the radical species. Indeed, if they find themselves confined in close proximity within the highly-ordered arrangement of chains (“cage”) in the crystalline domain it is easier for them to recombine rather than diffuse out and propagate, reducing the effective chain scissions and making it occur in a rather more controlled way within the crystalline domain. On the other hand, such a “cage effect” is certainly less expected for amorphous arrangements, where much more free space is left to the radicals to diffuse and propagate randomly through the overall polymer network [28,43].

Moreover, though the actual composition is left untouched (NMR in Figure S5 and XRD in Figure S6 in S.I.) detectable modifications are recorded also in the crystallinity of the two polymers upon irradiation. Indeed, the crystallinity degree of E is reduced and the actual well for the point of crystallization is shifted from 100 °C for the pristine polymer to 87 °C for the ionized one. Such changes are not easy to detect for the case of P, as already in its pristine appearance, it presents an amorphous nature, whose DSC curve does not present any noticeable wells or peaks (Figure 3).

### 3.4. Mathematical Modelling

Electron beam sterilization not only alters the initial polymer properties, but also accelerates the degradation rate. This cannot be disregarded, because a faster decay of the molecular weight reduces the time span where a device can assure the desired mechanical properties. Therefore, a comprehensive overview of the consequences of electron beam sterilization should couple the analysis of the detrimental effects on raw material as well as its accelerated degradation. 

For this purpose, mathematical modeling emerges as a useful tool that can provide a quantitative estimation of the molecular weight decay. In this framework, the model proposed by Perale et al. [44] and Casalini et al. [45] has been chosen for the comprehensive description of the involved phenomena (hydrolysis, autocatalysis, and transport phenomena) and its validated results. Details are extensively discussed in previous papers [44,45], but model formulation is here summarized for the sake of completeness. 

The model is based on population balances, where a mass balance for a polymer chain with *n* repeating units is written. Since it is necessary to write an equation for each considered chain length value, this approach would imply a large number of differential equation to be solved (about 10^5^). In order to reduce the computational effort, the method of the statistical moment is employed [46], allowing to reduce the large number of population balance equations to three equations, which accounts for the time and spatial evolution of the statistical moments of the first three orders. 

The generic *j*-th order moment μ_j_ is defined as follows:

(1) $$\;\mu_{j}=\overset{\infty }{\underset{n=1}{\sum }}n^{j}C_{n}\;$$

where *n* is chain length and *C*_n_ is the concentration of a polymer chain with *n* repeating units. 

Under the assumption that only oligomers up to nonamers can diffuse through polymer matrix, model is composed by a system of partial differential equations that account for time and spatial evolution of monomer (Equation (2)), water (Equation (3)), oligomers (Equation (4)) and statistical moments of zero-th, first and second order (Equations (5)–(7)):

(2) $$\;\frac{\partial C_{M}}{\partial t}=\nabla \left(D_{M}\nabla C_{M}\right)+2k_{d}C_{W}\left(\mu_{0}-C_{M}\right)\mu_{0}\;$$

(3) $$\;\frac{\partial C_{W}}{\partial t}=\nabla \left(D_{W}\nabla C_{W}\right)-k_{d}C_{W}\left(\mu_{1}-\mu_{0}\right)\mu_{0}\;$$

(4) $$\;\frac{\partial C_{n}}{\partial t}=\nabla (D_{\mathrm{olig}}\nabla C_{n})+2k_{d}C_{W}(\mu_{0}-\overset{n}{\underset{j=1}{\sum }}C_{j})\mu_{0}-\left(n-1\right)k_{d}C_{W}C_{n}\mu_{0}\;\;\;\;\;2\;\leq \;n\;\leq \;9\;$$

(5) $$\;\frac{\partial \mu_{0}}{\partial t}=\overset{9}{\underset{j=1}{\sum }}\nabla \left(D_{j}\nabla C_{j}\right)+k_{d}C_{W}\left(\mu_{1}-\mu_{0}\right)\mu_{0}\;$$

(6) $$\;\frac{\partial \mu_{1}}{\partial t}=\overset{9}{\underset{j=1}{\sum }}j\times \nabla \left(D_{j}\nabla C_{j}\right)\;$$

(7) $$\;\frac{\partial \mu_{2}}{\partial t}=\overset{9}{\underset{j=1}{\sum }}j^{2}\cdot \nabla \left(D_{j}\nabla C_{j}\right)+\frac{k_{d}C_{W}\mu_{0}}{3}\left(\mu_{1}-2\frac{\mu_{2}^{2}}{\mu_{1}}+\frac{\mu_{2}\mu_{1}}{\mu_{0}}\right)\;$$

where *C*_M_ is monomer concentration, *D*_M_ is monomer diffusion coefficient, *k*_d_ is degradation kinetic constant, *C*_W_ is water concentration, *D*_W_ is water diffusion coefficient, *C*_n_ is the concentration of an oligomer with n repeating units and *D*_olig_ is oligomer diffusion coefficient. 

The model takes into account the increase of diffusivity due to degradation (chain scissions open new and wider diffusive path) through the following expression:

(8) $$\;D_{i}=D_{i}^{0}\exp\left[2.5{\left(1-\frac{M_{n}\left(t,x\right)}{M_{n}\left(t=0\right)}\right)}^{0.5}\right]\;\;\;\;\;\;\;\;\;\;\;\;i\;=\;\mathrm{monomer},\;\mathrm{oligomer},\;\mathrm{water}\;$$

where *D*_i_^0^ is the diffusion coefficient of the *i*-th species before degradation onset, and *x* is a generic spatial coordinate.

The average properties of interest, such as number average molecular weight *M*_n_, weight average molecular weight *M*_w_ and polydispersity *PD* can be easily calculated starting from the statistical moments:

(9) $$\;M_{n}=\frac{\mu_{1}}{\mu_{0}}M_{\mathrm{mon}}\;$$

(10) $$\;M_{w}=\frac{\mu_{2}}{\mu_{1}}M_{\mathrm{mon}}\;$$

(11) $$\;PD=\frac{\mu_{2}\mu_{0}}{\mu_{1}^{2}}\;$$

where *M*_mon_ is the molecular weight of the repeating unit.

Model equations are here written in their general form, but only one spatial coordinate (the characteristic diffusion length) is usually considered in the Laplacian term. The system of partial differential equations constituted by Equations (2)–(7) is solved through the method of lines: spatial derivatives are approximated through a finite difference scheme (centered formulation) and the resulting system of ordinary differential equations is numerically integrated by means of the *ode15s* algorithm implemented in MATLAB.

The analysis has been performed starting from experimental data taken from Loo et al. [26], chosen as a reference case for their exhaustiveness. In particular, Loo and coworkers studied the influence of the irradiation dose on the initial properties and the hydrolytic degradation of the electron beam-irradiated films of polylactic acid. 

The mathematical model has been used in order to compute a degradation kinetic constant, which offers a quantitative estimation of the degradation enhancement provided by electron beam sterilization; film thickness has been considered as characteristic diffusion length. 

Kinetic constants have been obtained through fitting of experimental data, in order to best reproduce the decay of the number average molecular weight over time; data fitting has been carried out by means of lsqnonlin algorithm implemented in MATLAB. Model input parameters are summarized in Table 2.

**Figure 5:**
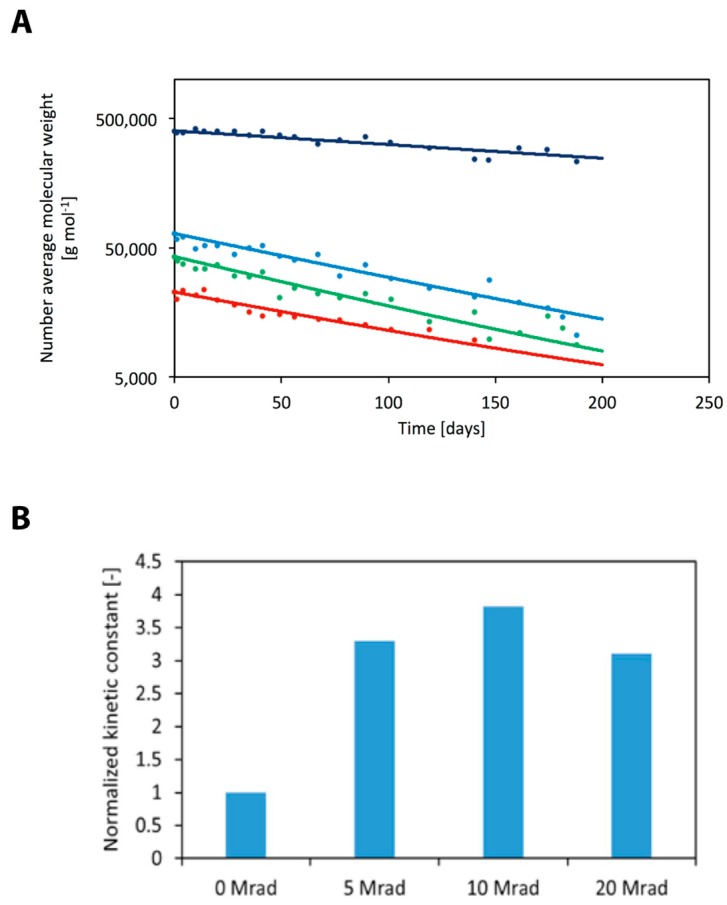
Comparison between model predictions (continuous line) and experimental data (filled circles) taken from Loo et al. [26] for different radiation doses (**A**). Normalized degradation kinetic constants as a function of radiation dose. Values have been normalized with respect to the kinetic constant of the non-irradiated polymer (**B**).

Initial conditions and kinetic constants are summarized in Table 3, while a comparison between model results and experimental data is shown in Figure 5A.

Model best fitting is in good agreement with the experimental data, thus confirming the reliability of the chosen approach. The obtained kinetic constants are listed in Table 3, while the normalized values are shown in Figure 5B. In particular, values have been normalized, choosing the non-irradiated polymer as a reference value. In primis, data confirm that electron beam sterilization accelerates polymer degradation, since it reduces the initial molecular weight through chain scission. According to model results, hydrolysis is about 3.5 times faster for the irradiated polymer; in addition, degradation enhancement does not depend on radiation dose, since the values of the kinetic constants are close to each other. 

This analysis highlights that, for an optimal device design, the initial molecular weight drop due to irradiation must be coupled with the enhanced degradation in order to identify the most suitable polymer. In other words, the choice of the raw material (i.e., before sterilization and processing) should take into account that the initial molecular weight will be not only reduced by processing and sterilization but will also decay faster. This is essential for those applications where the device must assure mechanical stability in a determined time span.

## 4. Conclusions

In this work we presented a case study for showing a possible methodologic approach for the selection of appropriate biodegradable polymers in product formulation for medical devices manufacturing. In particular, the major effect of the finished product terminal sterilization is discussed and a comparison between the EtO and e-beam treatments is presented for two apparently similar poly(l-lactide-*co*-ε-caprolactone) base materials with identical composition (70% PLA and 30% PCL). 

Specifically, the major difference resembles in the degree of crystallinity of the aforementioned polymer: E has a relatively high degree of 65% whereas P has a rather amorphous structure. Once sterilized, independently on the implied sterilization protocol, both polymers exhibited degradation. Actually, the EtO exposition minimally affected the initial characteristics (in terms of *M*_w_, *PD* and crystallinity), whereas major differences before and after the treatment was observed for e-beam irradiation. Indeed, a major reduction in molecular weight was observed for both polymers. Additionally, the different chains arrangement led to different responses to the treatment. Indeed, even if polymer P degraded apparently less than polymer E, the chain length distribution considerably broadened. This would reflect in a more uncontrolled change in the desired initial properties selected during product formulation and design. Moreover, irradiation has also a major effect on the degradation kinetic of polymers. This effect has been studied through a mathematical model, which allowed to identify that hydrolysis is about 3.5 times faster for the irradiated polymer than the pristine one, remarkably not being dependent on the radiation dose. 

As a conclusion, we believe that the presented example might be very useful in the development of bioresorbable devices. As a matter of fact, intrinsic response of the material upon the overall post-processing steps might be understood only upon a deep and appropriate characterization of the starting polymers, even when they are nominally similar. 

## Figures and Tables

**Table 1:** Data obtained by GPC, Nuclear Magnetic Resonance (^1^H-NMR), differential scanning calorimetry (DSC), and X-ray diffraction (XRD) of the polymeric samples.

	GPC			^1^H-NMR		DSC	XRD	
# Sample	*M*_w_ (Da)	*M*_n_ (Da)	*PD* (−)	CL (% g·g_tot_^−1^)	Crystallinity (%)	Crystallinity (%)	FWHM (°)	*L*_hkl_ (A)
E	119,600	85,660	1.12	25.4	61.1	65	4.5	0.308
P	172,750	123,800	1.6	25.5	36.1	35.2	7.5	0.185
E EtO	96,000	78,700	1.2	25	59.6	60	4.5	0.308
P EtO	159,400	97,300	1.64	25.7	34.3	35	7.5	0.185
E e-beam	51,800	43,660	1.3	25.6	60.6	59.2	4.5	0.317
P e-beam	133,300	64,100	2.08	25.4	35.5	32.1	7.5	0.185

**Table 2:** Model input parameters.

**Film thickness (μm)**	55
***M*_mon_ (g·mol^−1^)**	90.08
***ρ_pol_***	1.2
***D*_M_^0^ (cm^2^·s^−1^)**	10^−10^
***D*_olig_^0^ (cm^2^·s^−1^)**	10^−10^
***D*_W_^0^ (cm^2^·s^−1^)**	10^−8^

**Table 3:** Initial properties of the polymer analyzed through mathematical modeling and degradation kinetic constants obtained through experimental data fitting.

Radiation Dose (Mrad)	Number Average Molecular Weight (g·mol^−1^)	Polydispersity (−)	Degradation Constant (cm^6^·mol^−2^·s^−1^)
0	406,000	1.60	3.85 × 10^−5^
5	64,700	1.68	1.27 × 10^−4^
10	43,200	1.73	1.47 × 10^−4^
20	23,100	1.76	1.21 × 10^−4^

## Supplementary Materials

The following are available online at http://www.mdpi.com/2073-4360/10/8/851/s1, Figure S1: NMR spectra of P and E samples, Figure S2: XRD spectra of P (blue line) and E (red line), Figure S3: NMR spectra of P and E after EtO, Figure S4: XRD spectra of P (blue line) and E (red line) after EtO, Figure S5: NMR spectra of P and E after electron beam irradiation, Figure S6: XRD spectra of P and E after electron beam irradiation.
