# Supplementary material for: A Methodologic Approach for the Selection of Bio-Resorbable Polymers in the Development of Medical Devices: The Case of Poly(l-lactide-co-ε-caprolactone)
Source: Polymers (Basel). 2018 Aug 1;10(8):851. doi: 10.3390/polym10080851 (PMC6403915; doi:10.3390/polym10080851)
Supplement: Supplementary file 1 [file polymers-10-00851-s001.pdf]

polymers

MDPI

Supporting information

A Methodologic Approach for the Selection of Bio-Resorbable Polymers in the Development of Medical Devices: The Case of Poly(L-lactide-*co*-ε-caprolactone)

Alberto Cingolani [1,2], Tommaso Casalini [1,3], Stefano Caimi [1], Antoine Klaue [1], Mattia Sponchioni [4], Filippo Rossi [4,*] and Giuseppe Perale [2,3,*]

1. Institute for Chemical and Bioengineering, Department of Chemistry and Applied Bioscience ETH Zurich, Vladimir-Prelog-Weg 1-5/10, 8093 Zürich, Switzerland; alberto.cingolani@chem.ethz.ch (A.C.); tommaso.casalini@chem.ethz.ch (T.C.); stefano.caimi@chem.ethz.ch (S.C.); antoine.klaue@chem.ethz.ch (A.K.)
2. Industrie Biomediche Insubri SA (IBI), Via Cantonale 67, 6805 Mezzovico-Vira, Switzerland
3. Biomaterials Laboratory, Institute for Mechanical Engineering and Materials Technology, SUPSI—University of Applied Sciences and Arts of Southern Switzerland, Via Cantonale 2C, Galleria 2, 6928 Manno, Switzerland
4. Department of Chemistry, Materials and Chemical Engineering "G. Natta", Politecnico di Milano, 20100 Milan, Italy; mattia.sponchioni@polimi.it

* Correspondence: filippo.rossi@polimi.it (F.R.); giuseppe.perale@supsi.ch (G.P.); Tel: +39-02-2399-3145 (F.R.); +41-58-666-66-41 (G.P.)

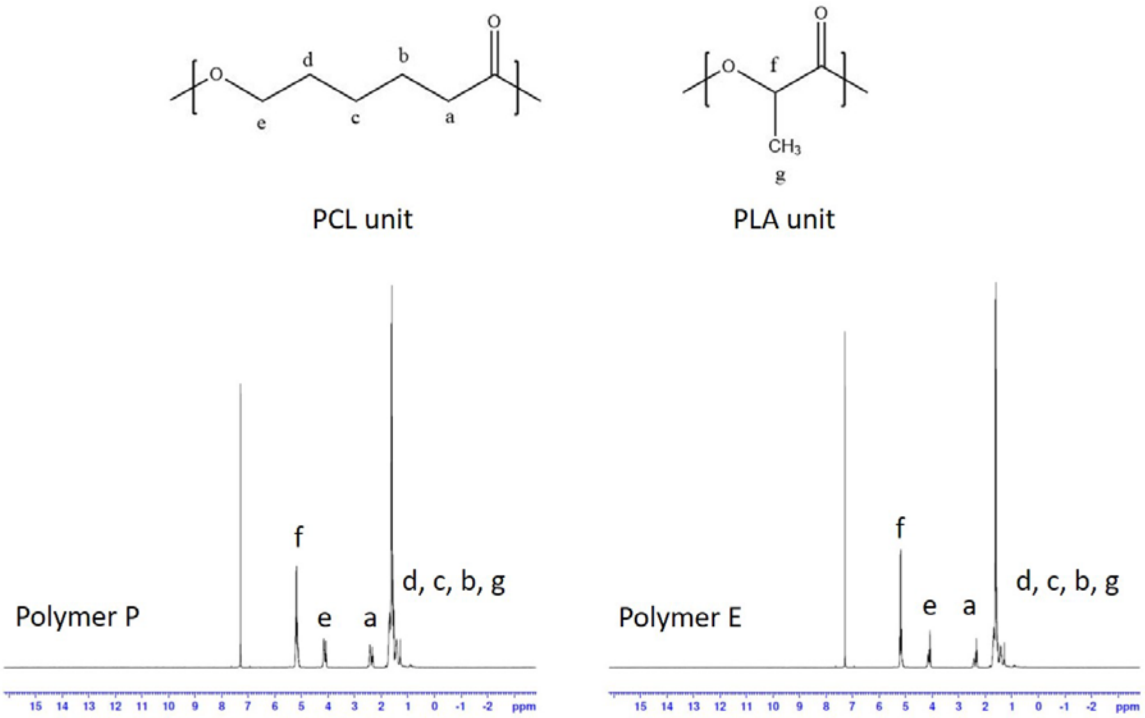

Figure S1. NMR spectra of P and E samples.

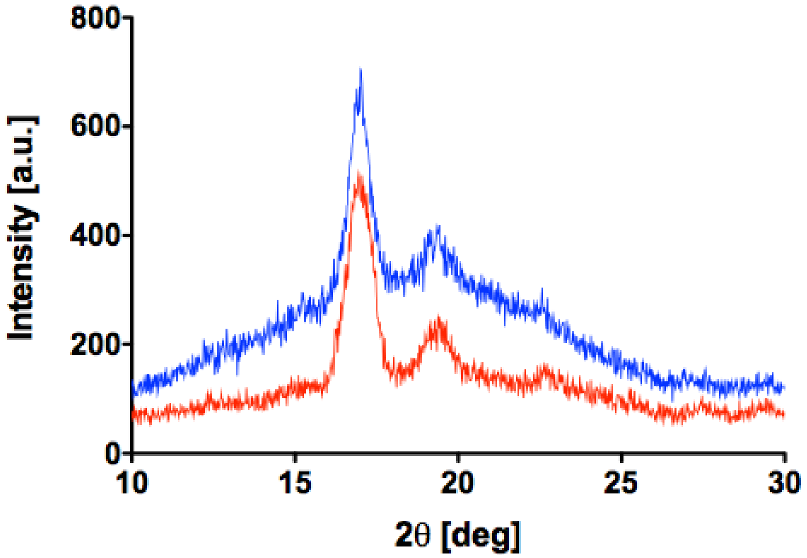

Figure S2. XRD spectra of P (blue line) and E (red line).

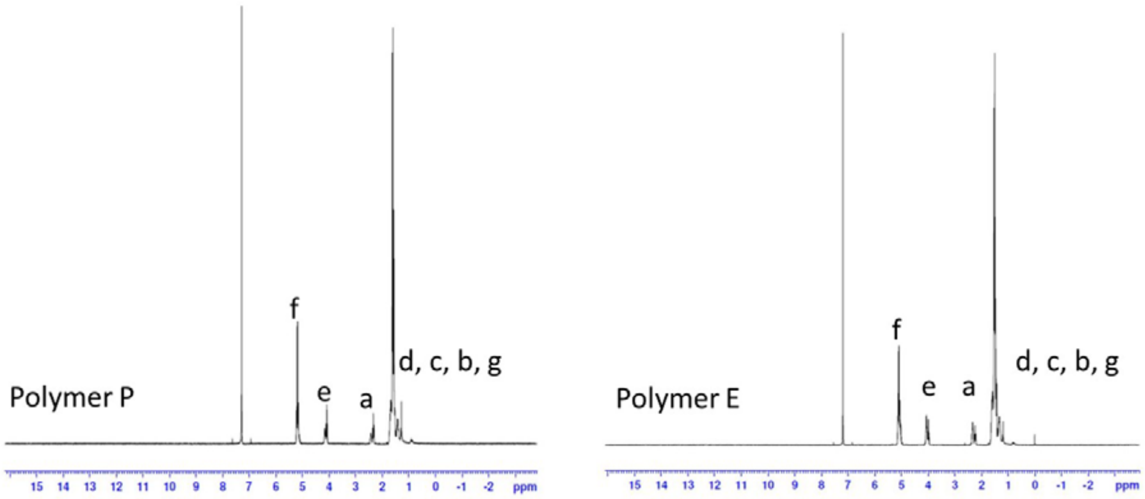

Figure S3. NMR spectra of P and E after EtO.

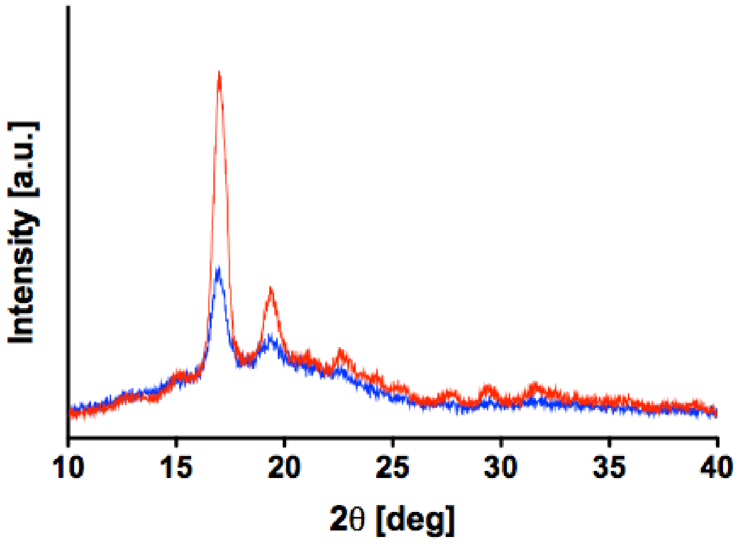

Figure S4. XRD spectra of P (blue line) and E (red line) after EtO.

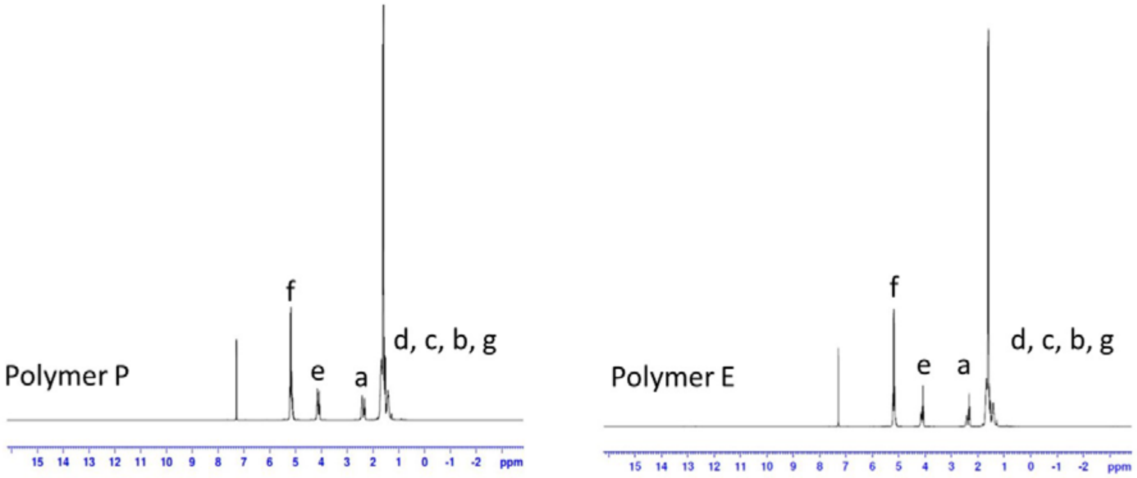

Figure S5. NMR spectra of P and E after electron beam irradiation.

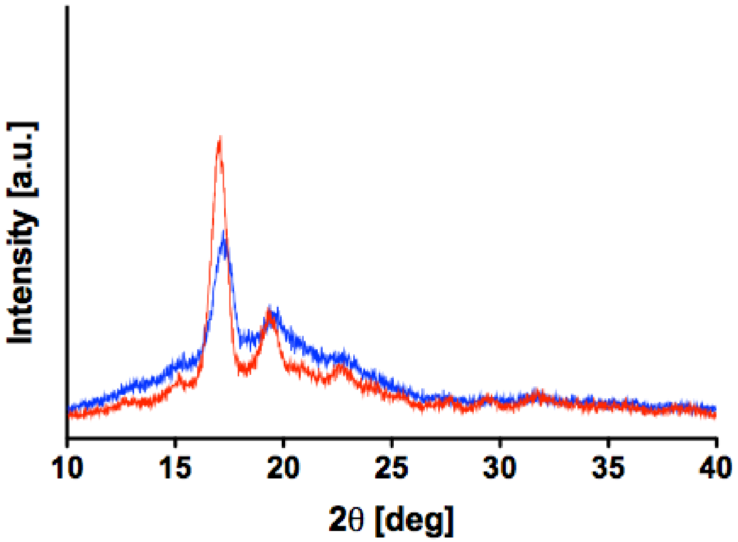

Figure S6. XRD spectra of P and E after electron beam irradiation.

© 2018 by the authors. Submitted for possible open access publication under the terms and conditions of the Creative Commons Attribution (CC BY) license (http://creativecommons.org/licenses/by/4.0/).
